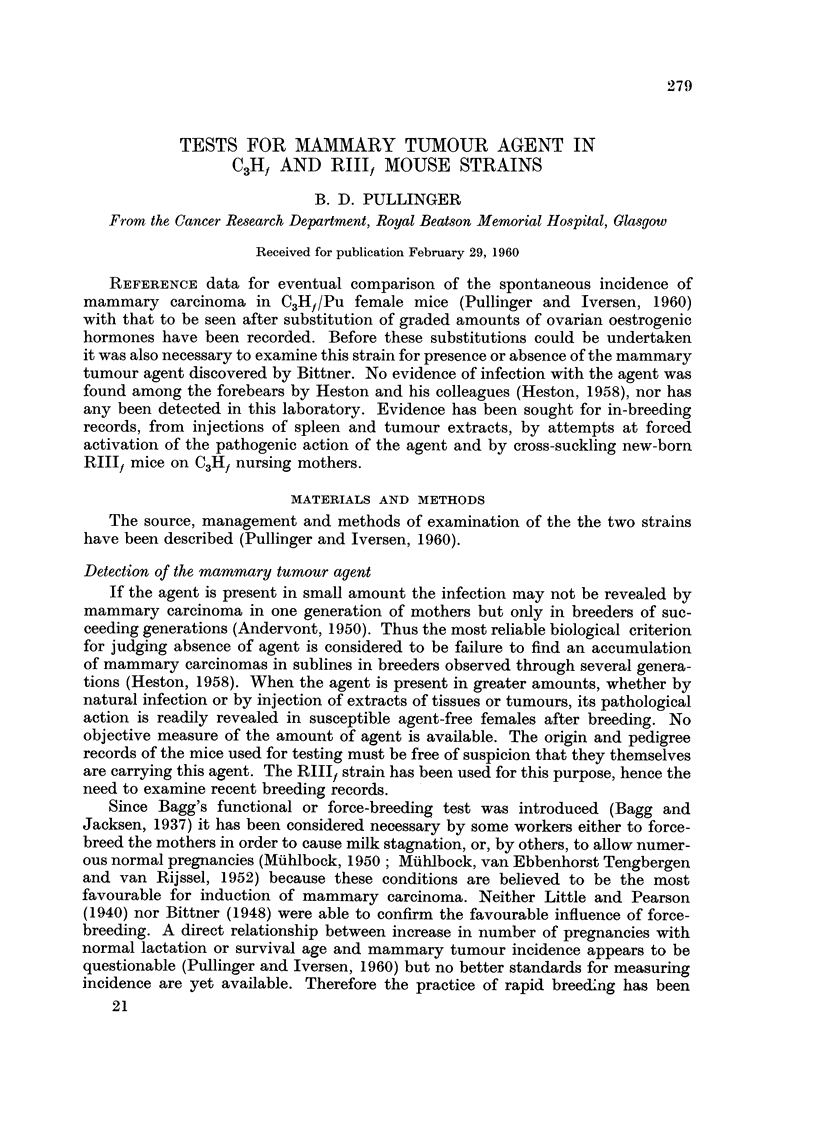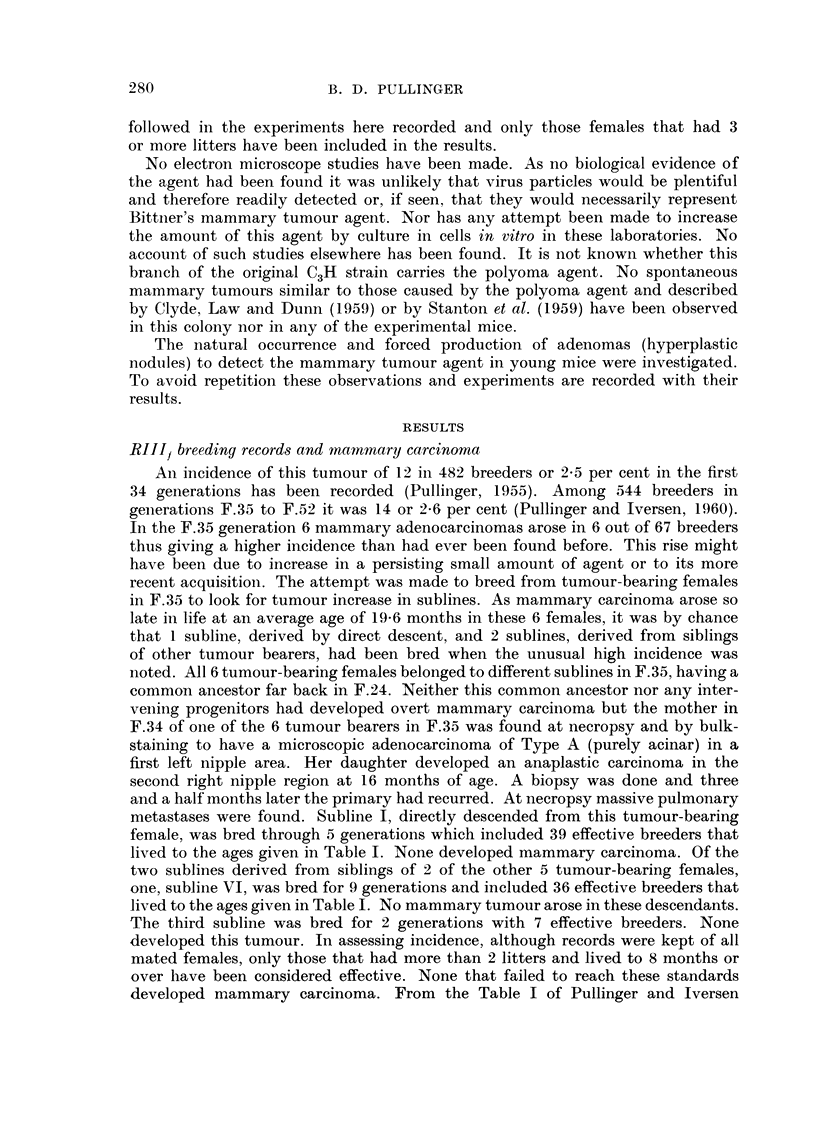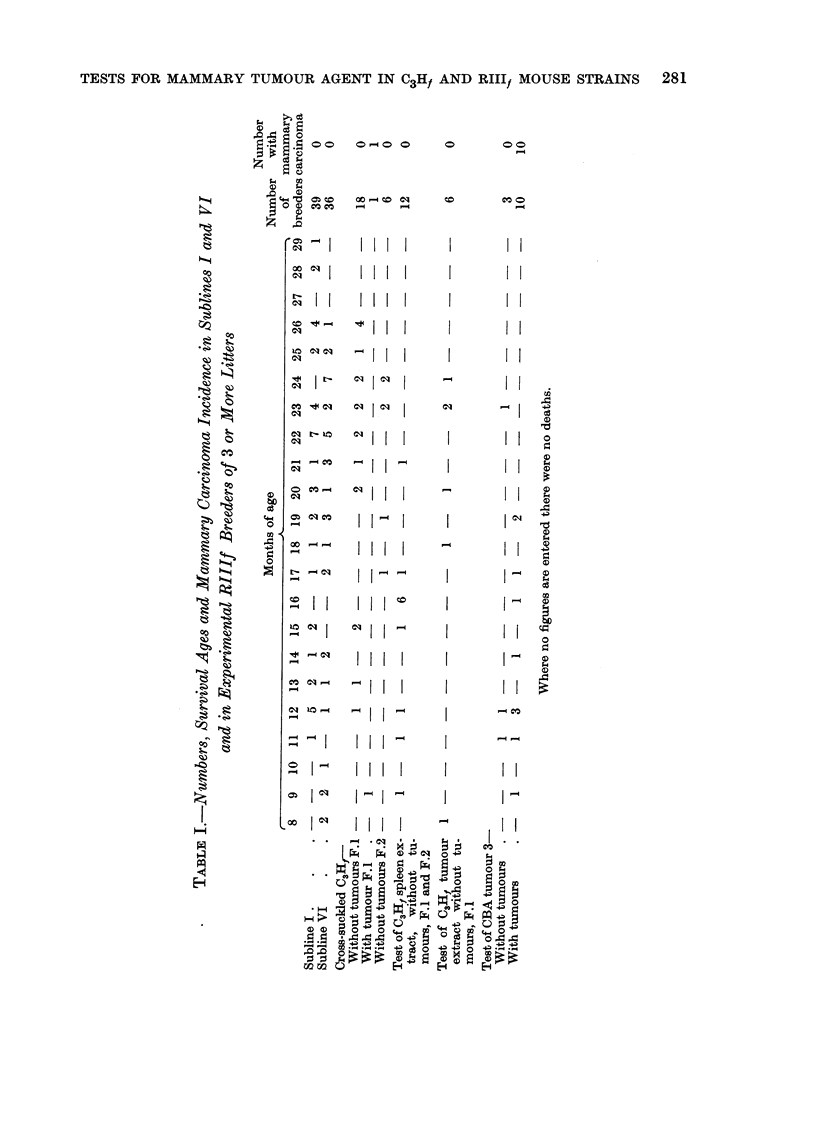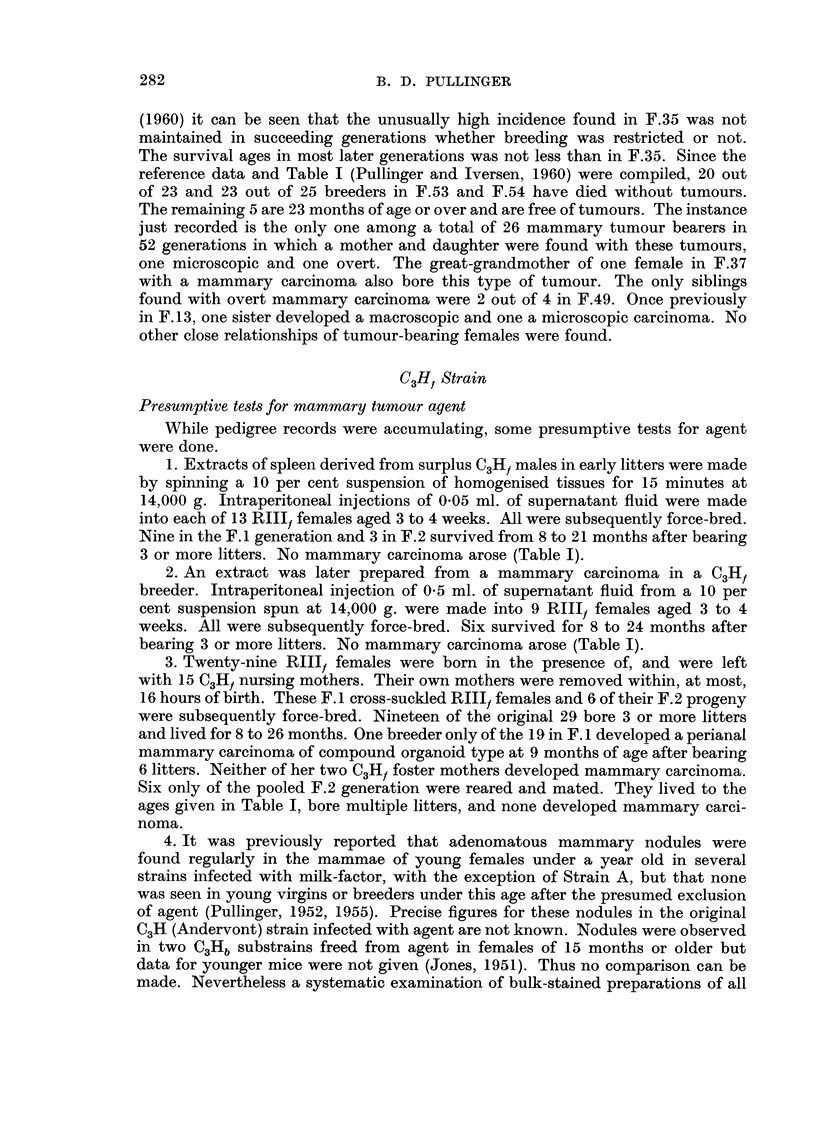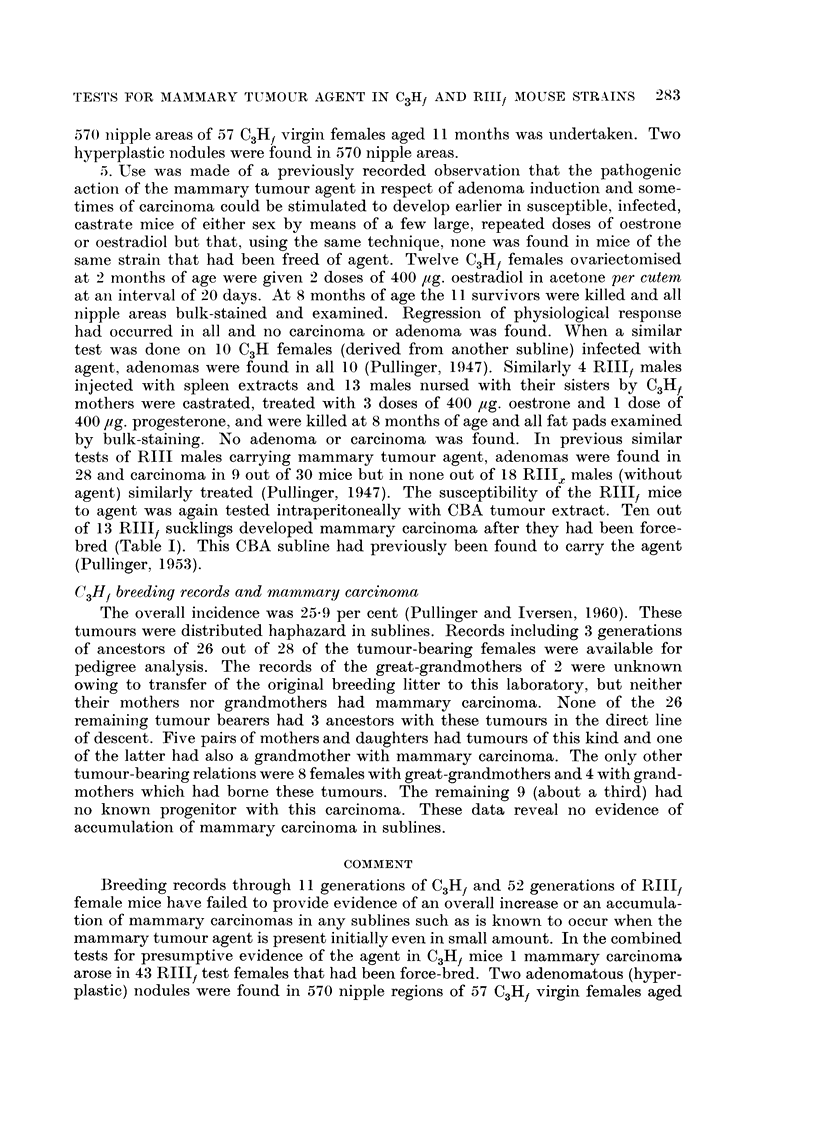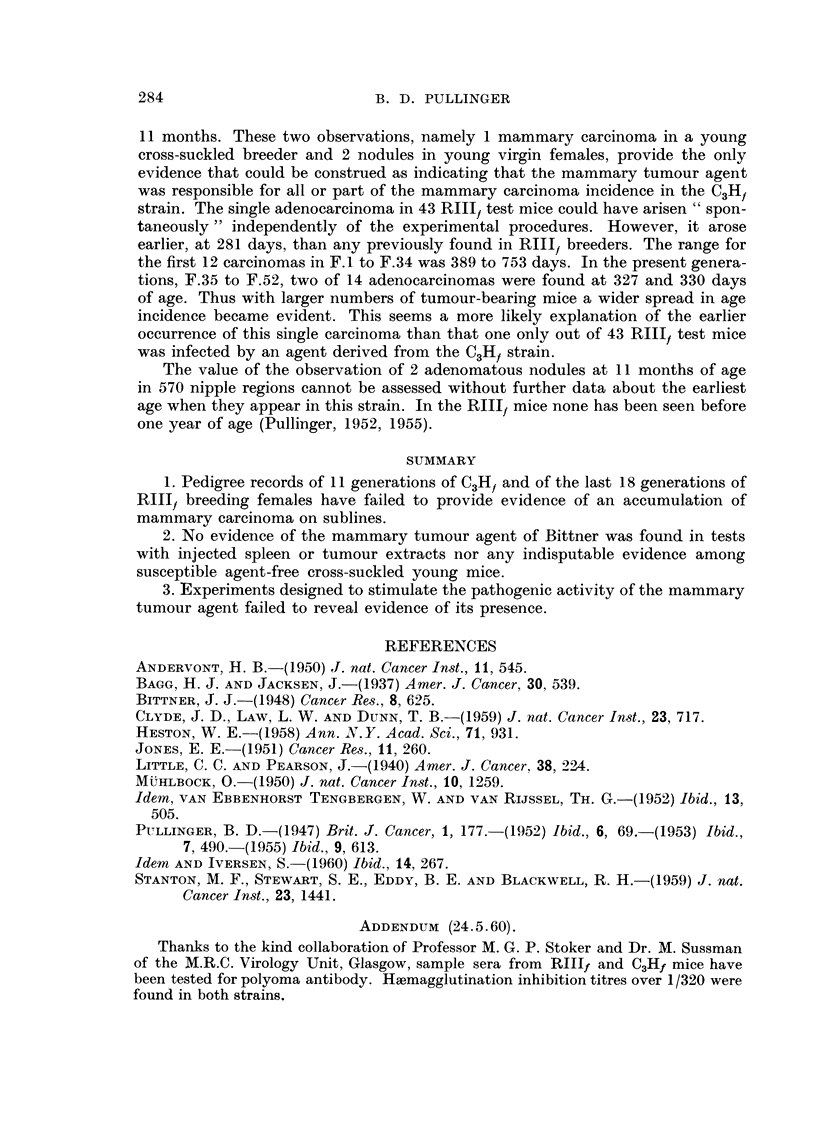# Tests for Mammary Tumour Agent in C_3_H_f_ and RIII_f_ Mouse Strains

**DOI:** 10.1038/bjc.1960.32

**Published:** 1960-06

**Authors:** B. D. Pullinger


### TESTS FOR MAMMARY TUMOUR AGENT IN $C_3H_f$ AND $RIII_f$ MOUSE STRAINS

B. D. PULLINGER

*From the Cancer Research Department, Royal Beatson Memorial Hospital, Glasgow*

Received for publication February 29, 1960

REFERENCE data for eventual comparison of the spontaneous incidence of mammary carcinoma in $C_3H_f$/Pu female mice (Pullinger and Iversen, 1960) with that to be seen after substitution of graded amounts of ovarian oestrogenic hormones have been recorded. Before these substitutions could be undertaken it was also necessary to examine this strain for presence or absence of the mammary tumour agent discovered by Bittner. No evidence of infection with the agent was found among the forebears by Heston and his colleagues (Heston, 1958), nor has any been detected in this laboratory. Evidence has been sought for in-breeding records, from injections of spleen and tumour extracts, by attempts at forced activation of the pathogenic action of the agent and by cross-suckling new-born $RIII_f$ mice on $C_3H_f$ nursing mothers.

### MATERIALS AND METHODS

The source, management and methods of examination of the the two strains have been described (Pullinger and Iversen, 1960).

### *Detection of the mammary tumour agent*

If the agent is present in small amount the infection may not be revealed by mammary carcinoma in one generation of mothers but only in breeders of succeeding generations (Andervont, 1950). Thus the most reliable biological criterion for judging absence of agent is considered to be failure to find an accumulation of mammary carcinomas in sublines in breeders observed through several generations (Heston, 1958). When the agent is present in greater amounts, whether by natural infection or by injection of extracts of tissues or tumours, its pathological action is readily revealed in susceptible agent-free females after breeding. No objective measure of the amount of agent is available. The origin and pedigree records of the mice used for testing must be free of suspicion that they themselves are carrying this agent. The $RIII_f$ strain has been used for this purpose, hence the need to examine recent breeding records.

Since Bagg's functional or force-breeding test was introduced (Bagg and Jacksen, 1937) it has been considered necessary by some workers either to force-breed the mothers in order to cause milk stagnation, or, by others, to allow numerous normal pregnancies (Mühlbock, 1950 ; Mühlbock, van Ebbenhorst Tengbergen and van Rijssel, 1952) because these conditions are believed to be the most favourable for induction of mammary carcinoma. Neither Little and Pearson (1940) nor Bittner (1948) were able to confirm the favourable influence of force-breeding. A direct relationship between increase in number of pregnancies with normal lactation or survival age and mammary tumour incidence appears to be questionable (Pullinger and Iversen, 1960) but no better standards for measuring incidence are yet available. Therefore the practice of rapid breeding has been followed in the experiments here recorded and only those females that had 3 or more litters have been included in the results.

No electron microscope studies have been made. As no biological evidence of the agent had been found it was unlikely that virus particles would be plentiful and therefore readily detected or, if seen, that they would necessarily represent Bittner's mammary tumour agent. Nor has any attempt been made to increase the amount of this agent by culture in cells *in vitro* in these laboratories. No account of such studies elsewhere has been found. It is not known whether this branch of the original $C_3H$ strain carries the polyoma agent. No spontaneous mammary tumours similar to those caused by the polyoma agent and described by Clyde, Law and Dunn (1959) or by Stanton *et al.* (1959) have been observed in this colony nor in any of the experimental mice.

The natural occurrence and forced production of adenomas (hyperplastic nodules) to detect the mammary tumour agent in young mice were investigated. To avoid repetition these observations and experiments are recorded with their results.

### RESULTS

### *$RIII_f$ breeding records and mammary carcinoma*

An incidence of this tumour of 12 in 482 breeders or 2·5 per cent in the first 34 generations has been recorded (Pullinger, 1955). Among 544 breeders in generations F.35 to F.52 it was 14 or 2·6 per cent (Pullinger and Iversen, 1960). In the F.35 generation 6 mammary adenocarcinomas arose in 6 out of 67 breeders thus giving a higher incidence than had ever been found before. This rise might have been due to increase in a persisting small amount of agent or to its more recent acquisition. The attempt was made to breed from tumour-bearing females in F.35 to look for tumour increase in sublines. As mammary carcinoma arose so late in life at an average age of 19·6 months in these 6 females, it was by chance that 1 subline, derived by direct descent, and 2 sublines, derived from siblings of other tumour bearers, had been bred when the unusual high incidence was noted. All 6 tumour-bearing females belonged to different sublines in F.35, having a common ancestor far back in F.24. Neither this common ancestor nor any intervening progenitors had developed overt mammary carcinoma but the mother in F.34 of one of the 6 tumour bearers in F.35 was found at necropsy and by bulk-staining to have a microscopic adenocarcinoma of Type A (purely acinar) in a first left nipple area. Her daughter developed an anaplastic carcinoma in the second right nipple region at 16 months of age. A biopsy was done and three and a half months later the primary had recurred. At necropsy massive pulmonary metastases were found. Subline I, directly descended from this tumour-bearing female, was bred through 5 generations which included 39 effective breeders that lived to the ages given in Table I. None developed mammary carcinoma. Of the two sublines derived from siblings of 2 of the other 5 tumour-bearing females, one, subline VI, was bred for 9 generations and included 36 effective breeders that lived to the ages given in Table I. No mammary tumour arose in these descendants. The third subline was bred for 2 generations with 7 effective breeders. None developed this tumour. In assessing incidence, although records were kept of all mated females, only those that had more than 2 litters and lived to 8 months or over have been considered effective. None that failed to reach these standards developed mammary carcinoma. From the Table I of Pullinger and Iversen

TABLE I.—*Numbers, Survival Ages and Mammary Carcinoma Incidence in Sublines I and VI and in Experimental RIIIf Breeders of 3 or More Litters*

| | Months of age | | | | | | | | | | | | | | | | | | | | | | Number of breeders | Number with mammary carcinoma |
|---|---|---|---|---|---|---|---|---|---|---|---|---|---|---|---|---|---|---|---|---|---|---|---|---|
| | 8 | 9 | 10 | 11 | 12 | 13 | 14 | 15 | 16 | 17 | 18 | 19 | 20 | 21 | 22 | 23 | 24 | 25 | 26 | 27 | 28 | 29 | | |
| Subline I . . . | — | — | — | 1 | 5 | 2 | 1 | 2 | — | 1 | 1 | 2 | 3 | 1 | 7 | 4 | — | 2 | 4 | — | 2 | 1 | 39 | 0 |
| Subline VI . . | 2 | 2 | 1 | — | 1 | 1 | 2 | — | — | 2 | 1 | 3 | 1 | 3 | 5 | 2 | 7 | 2 | 1 | — | — | — | 36 | 0 |
| Cross-suckled $C_3H_f$— | | | | | | | | | | | | | | | | | | | | | | | | |
| Without tumours F.1 | — | — | — | — | 1 | 1 | — | 2 | — | — | — | — | 2 | 1 | 2 | 2 | 2 | 1 | 4 | — | — | — | 18 | 0 |
| With tumour F.1 . | — | 1 | — | — | — | — | — | — | — | — | — | — | — | — | — | — | — | — | — | — | — | — | 1 | 1 |
| Without tumours F.2 | — | — | — | — | — | — | — | — | — | 1 | — | 1 | — | — | — | 2 | 2 | — | — | — | — | — | 6 | 0 |
| Test of $C_3H_f$ spleen extract, without tumours, F.1 and F.2 | — | 1 | — | 1 | 1 | — | — | 1 | 6 | 1 | — | — | — | 1 | — | — | — | — | — | — | — | — | 12 | 0 |
| Test of $C_3H_f$ tumour extract without tumours, F.1 | 1 | — | — | — | — | — | — | — | — | — | 1 | — | 1 | — | — | 2 | 1 | — | — | — | — | — | 6 | 0 |
| Test of CBA tumour 3— | | | | | | | | | | | | | | | | | | | | | | | | |
| Without tumours . | — | — | — | 1 | 1 | — | — | — | — | — | — | — | — | — | — | 1 | — | — | — | — | — | — | 3 | 0 |
| With tumours . | — | 1 | — | 1 | 3 | — | 1 | — | 1 | 1 | — | 2 | — | — | — | — | — | — | — | — | — | — | 10 | 10 |

Where no figures are entered there were no deaths.

(1960) it can be seen that the unusually high incidence found in F.35 was not maintained in succeeding generations whether breeding was restricted or not. The survival ages in most later generations was not less than in F.35. Since the reference data and Table I (Pullinger and Iversen, 1960) were compiled, 20 out of 23 and 23 out of 25 breeders in F.53 and F.54 have died without tumours. The remaining 5 are 23 months of age or over and are free of tumours. The instance just recorded is the only one among a total of 26 mammary tumour bearers in 52 generations in which a mother and daughter were found with these tumours, one microscopic and one overt. The great-grandmother of one female in F.37 with a mammary carcinoma also bore this type of tumour. The only siblings found with overt mammary carcinoma were 2 out of 4 in F.49. Once previously in F.13, one sister developed a macroscopic and one a microscopic carcinoma. No other close relationships of tumour-bearing females were found.

### $C_3H_f$ *Strain*

*Presumptive tests for mammary tumour agent*

While pedigree records were accumulating, some presumptive tests for agent were done.

1. Extracts of spleen derived from surplus $C_3H_f$ males in early litters were made by spinning a 10 per cent suspension of homogenised tissues for 15 minutes at 14,000 g. Intraperitoneal injections of 0·05 ml. of supernatant fluid were made into each of 13 $RIII_f$ females aged 3 to 4 weeks. All were subsequently force-bred. Nine in the F.1 generation and 3 in F.2 survived from 8 to 21 months after bearing 3 or more litters. No mammary carcinoma arose (Table I).

2. An extract was later prepared from a mammary carcinoma in a $C_3H_f$ breeder. Intraperitoneal injection of 0·5 ml. of supernatant fluid from a 10 per cent suspension spun at 14,000 g. were made into 9 $RIII_f$ females aged 3 to 4 weeks. All were subsequently force-bred. Six survived for 8 to 24 months after bearing 3 or more litters. No mammary carcinoma arose (Table I).

3. Twenty-nine $RIII_f$ females were born in the presence of, and were left with 15 $C_3H_f$ nursing mothers. Their own mothers were removed within, at most, 16 hours of birth. These F.1 cross-suckled $RIII_f$ females and 6 of their F.2 progeny were subsequently force-bred. Nineteen of the original 29 bore 3 or more litters and lived for 8 to 26 months. One breeder only of the 19 in F.1 developed a perianal mammary carcinoma of compound organoid type at 9 months of age after bearing 6 litters. Neither of her two $C_3H_f$ foster mothers developed mammary carcinoma. Six only of the pooled F.2 generation were reared and mated. They lived to the ages given in Table I, bore multiple litters, and none developed mammary carcinoma.

4. It was previously reported that adenomatous mammary nodules were found regularly in the mammae of young females under a year old in several strains infected with milk-factor, with the exception of Strain A, but that none was seen in young virgins or breeders under this age after the presumed exclusion of agent (Pullinger, 1952, 1955). Precise figures for these nodules in the original $C_3H$ (Andervont) strain infected with agent are not known. Nodules were observed in two $C_3H_b$ substrains freed from agent in females of 15 months or older but data for younger mice were not given (Jones, 1951). Thus no comparison can be made. Nevertheless a systematic examination of bulk-stained preparations of all

570 nipple areas of 57 $C_3H_f$ virgin females aged 11 months was undertaken. Two hyperplastic nodules were found in 570 nipple areas.

5. Use was made of a previously recorded observation that the pathogenic action of the mammary tumour agent in respect of adenoma induction and sometimes of carcinoma could be stimulated to develop earlier in susceptible, infected, castrate mice of either sex by means of a few large, repeated doses of oestrone or oestradiol but that, using the same technique, none was found in mice of the same strain that had been freed of agent. Twelve $C_3H_f$ females ovariectomised at 2 months of age were given 2 doses of 400 $\mu$g. oestradiol in acetone *per cutem* at an interval of 20 days. At 8 months of age the 11 survivors were killed and all nipple areas bulk-stained and examined. Regression of physiological response had occurred in all and no carcinoma or adenoma was found. When a similar test was done on 10 $C_3H$ females (derived from another subline) infected with agent, adenomas were found in all 10 (Pullinger, 1947). Similarly 4 $RIII_f$ males injected with spleen extracts and 13 males nursed with their sisters by $C_3H_f$ mothers were castrated, treated with 3 doses of 400 $\mu$g. oestrone and 1 dose of 400 $\mu$g. progesterone, and were killed at 8 months of age and all fat pads examined by bulk-staining. No adenoma or carcinoma was found. In previous similar tests of RIII males carrying mammary tumour agent, adenomas were found in 28 and carcinoma in 9 out of 30 mice but in none out of 18 $RIII_x$ males (without agent) similarly treated (Pullinger, 1947). The susceptibility of the $RIII_f$ mice to agent was again tested intraperitoneally with CBA tumour extract. Ten out of 13 $RIII_f$ sucklings developed mammary carcinoma after they had been force-bred (Table I). This CBA subline had previously been found to carry the agent (Pullinger, 1953).

*$C_3H_f$ breeding records and mammary carcinoma*

The overall incidence was 25·9 per cent (Pullinger and Iversen, 1960). These tumours were distributed haphazard in sublines. Records including 3 generations of ancestors of 26 out of 28 of the tumour-bearing females were available for pedigree analysis. The records of the great-grandmothers of 2 were unknown owing to transfer of the original breeding litter to this laboratory, but neither their mothers nor grandmothers had mammary carcinoma. None of the 26 remaining tumour bearers had 3 ancestors with these tumours in the direct line of descent. Five pairs of mothers and daughters had tumours of this kind and one of the latter had also a grandmother with mammary carcinoma. The only other tumour-bearing relations were 8 females with great-grandmothers and 4 with grandmothers which had borne these tumours. The remaining 9 (about a third) had no known progenitor with this carcinoma. These data reveal no evidence of accumulation of mammary carcinoma in sublines.

### COMMENT

Breeding records through 11 generations of $C_3H_f$ and 52 generations of $RIII_f$ female mice have failed to provide evidence of an overall increase or an accumulation of mammary carcinomas in any sublines such as is known to occur when the mammary tumour agent is present initially even in small amount. In the combined tests for presumptive evidence of the agent in $C_3H_f$ mice 1 mammary carcinoma arose in 43 $RIII_f$ test females that had been force-bred. Two adenomatous (hyperplastic) nodules were found in 570 nipple regions of 57 $C_3H_f$ virgin females aged

11 months. These two observations, namely 1 mammary carcinoma in a young cross-suckled breeder and 2 nodules in young virgin females, provide the only evidence that could be construed as indicating that the mammary tumour agent was responsible for all or part of the mammary carcinoma incidence in the $C_3H_f$ strain. The single adenocarcinoma in 43 $RIII_f$ test mice could have arisen " spontaneously " independently of the experimental procedures. However, it arose earlier, at 281 days, than any previously found in $RIII_f$ breeders. The range for the first 12 carcinomas in F.1 to F.34 was 389 to 753 days. In the present generations, F.35 to F.52, two of 14 adenocarcinomas were found at 327 and 330 days of age. Thus with larger numbers of tumour-bearing mice a wider spread in age incidence became evident. This seems a more likely explanation of the earlier occurrence of this single carcinoma than that one only out of 43 $RIII_f$ test mice was infected by an agent derived from the $C_3H_f$ strain.

The value of the observation of 2 adenomatous nodules at 11 months of age in 570 nipple regions cannot be assessed without further data about the earliest age when they appear in this strain. In the $RIII_f$ mice none has been seen before one year of age (Pullinger, 1952, 1955).

### SUMMARY

1. Pedigree records of 11 generations of $C_3H_f$ and of the last 18 generations of $RIII_f$ breeding females have failed to provide evidence of an accumulation of mammary carcinoma on sublines.

2. No evidence of the mammary tumour agent of Bittner was found in tests with injected spleen or tumour extracts nor any indisputable evidence among susceptible agent-free cross-suckled young mice.

3. Experiments designed to stimulate the pathogenic activity of the mammary tumour agent failed to reveal evidence of its presence.

### REFERENCES

ANDERVONT, H. B.—(1950) *J. nat. Cancer Inst.*, **11**, 545.
BAGG, H. J. AND JACKSEN, J.—(1937) *Amer. J. Cancer*, **30**, 539.
BITTNER, J. J.—(1948) *Cancer Res.*, **8**, 625.
CLYDE, J. D., LAW, L. W. AND DUNN, T. B.—(1959) *J. nat. Cancer Inst.*, **23**, 717.
HESTON, W. E.—(1958) *Ann. N.Y. Acad. Sci.*, **71**, 931.
JONES, E. E.—(1951) *Cancer Res.*, **11**, 260.
LITTLE, C. C. AND PEARSON, J.—(1940) *Amer. J. Cancer*, **38**, 224.
MÜHLBOCK, O.—(1950) *J. nat. Cancer Inst.*, **10**, 1259.
*Idem*, VAN EBBENHORST TENGBERGEN, W. AND VAN RIJSSEL, TH. G.—(1952) *Ibid.*, **13**, 505.
PULLINGER, B. D.—(1947) *Brit. J. Cancer*, **1**, 177.—(1952) *Ibid.*, **6**, 69.—(1953) *Ibid.*, **7**, 490.—(1955) *Ibid.*, **9**, 613.
*Idem* AND IVERSEN, S.—(1960) *Ibid.*, **14**, 267.
STANTON, M. F., STEWART, S. E., EDDY, B. E. AND BLACKWELL, R. H.—(1959) *J. nat. Cancer Inst.*, **23**, 1441.

### ADDENDUM (24.5.60).

Thanks to the kind collaboration of Professor M. G. P. Stoker and Dr. M. Sussman of the M.R.C. Virology Unit, Glasgow, sample sera from $RIII_f$ and $C_3H_f$ mice have been tested for polyoma antibody. Hæmagglutination inhibition titres over 1/320 were found in both strains.